# Involvement of Activation of PKR in HBx-siRNA-Mediated Innate Immune Effects on HBV Inhibition

**DOI:** 10.1371/journal.pone.0027931

**Published:** 2011-12-08

**Authors:** Qiuju Han, Cai Zhang, Jian Zhang, Zhigang Tian

**Affiliations:** 1 Institute of Immunopharmacology and Immunotherapy, School of Pharmaceutical Sciences, Shandong University, Jinan, China; 2 Department of Immunology, School of Life Sciences, University of Science and Technology of China, Hefei, China; Institut Pasteur, France

## Abstract

RNA interference (RNAi) of virus-specific genes offers the possibility of developing a new anti-hepatitis B virus (anti-HBV) therapy. Recent studies have revealed that siRNAs can induce an innate immune response *in vitro* and *in vivo*. Here, HBVx (HBx) mRNA expression and HBV replication were significantly inhibited, followed by the enhancement of expression of type I interferons (IFNs), IFN-stimulated genes (ISG15 and ISG56) and proinflammatory cytokines after HepG2.2.15 cells were transfected with chemically synthesized HBx-siRNAs. Transfection with HBx-siRNAs also significantly increased expression of dsRNA-dependent protein kinase R (PKR) in HepG2.2.15 cells, followed by activation of downstream signaling events such as eukaryotic initiation factor 2α (eIF2-α). In PKR-over-expressing HepG2.2.15 cells, HBx-siRNAs exerted more potent inhibitory effects on HBV replication and greater production of type I IFNs. By contrast, the inhibitory effect of HBx-siRNAs on HBV replication was attenuated when PKR was inhibited or silenced, demonstrating that HBx-siRNAs greatly promoted PKR activation, leading to the higher production of type I IFN. Therefore, we concluded that PKR is involved in the innate immune effects mediated by HBx-siRNAs and further contributes to HBV inhibition. The bifunctional siRNAs with both gene silencing and innate immune activation properties may represent a new potential strategy for treatment of HBV.

## Introduction

Hepatitis B virus (HBV) infection is an important disease with 400 million HBV carriers worldwide. HBV infection may cause cirrhosis and hepatocellular carcinoma (HCC) and HBV-induced diseases cause 1 million deaths annually [1]. Recent studies have shown that RNA interference (RNAi) is a process whereby double-stranded RNA (dsRNA) induces a sequence-specific post-transcriptional silencing of homologous genes. This process is an evolutionarily conserved surveillance mechanism mediated by small interfering RNAs (siRNAs) of the length of 21–23 nucleotides. In the natural RNAi pathway, siRNAs are derived from the processing of long dsRNAs by the nuclease Dicer into discrete 21-mers [2], [3]. A number of recent studies have demonstrated that chemically synthesized siRNAs or vector-expressed shRNA targeting HBV elements inhibit HBV replication [4], [5], [6], [7].

It is noted that intracellular delivery of siRNA could activate immune system and induce the production of cytokines both *in vivo* and *in vitro*. The response by RNA may be induced by three classes of viral pattern recognition receptors (PRRs): the Toll-like receptors (TLRs), the RIG-like helicase (RLH) receptors such as RIG-I and MDA-5, nucleotide-binding domain (NBD)- and leucine-rich-region (LRR)-containing family of cytoplasmic proteins (known as NLRs) [8]. RIG-I is the key sensor of negative strand RNA viruses in the cytosol of cells [9], [10], recognizing RNA with a triphosphate group at the 5′-end in a sequence-independent manner. Some synthetic and natural RNAs (e.g. poly I:C, virus genomes, virus replication intermediates, viral transcripts, or self-RNA cleaved by RNase L) also serve as RIG-I agonists [9], [10], [11], [12], [13]. The natural ligand for MDA-5 remains to be identified, but long poly I:C can serve as an artificial agonist for this RLH [14]. TLR3 is engaged specifically by dsRNA, which is present either in viral genomes or generated after viral replication, and is involved in the induction of type I IFN responses. Indeed, a group of siRNAs stimulated monocytes or dendritic cells to produce proinflammatory cytokines and type I IFNs [15], [16]. In addition, some studies have demonstrated that immune recognition of siRNA is sequence dependent and likely involves signaling through the endosomal TLR7 pathway [17], [18].

The dsRNA-dependent protein kinase R (PKR), acting as a cytoplasmic RNA sensor, can also recognize molecular patterns in RNA and thus differentiate self from non-self. During viral infection, PKR binds viral dsRNA, autophosphorylates and subsequently phosphorylates the alpha subunit of translation initiation factor 2 (eIF2-α) [19], [20]. Several reports reported that siRNAs can activate PKR signal pathway in a sequence independent manner, particularly when they are combined with lipids, during *in vitro* cell transfection [21], [22], [23]. The mechanisms that enable cells to sense and respond to dsRNA are not completely understood.

The HBV genome consists of four open reading frames (ORFs) with overlapping sequences. The HBVx (HBx) gene is essential for HBV viral infection and plays an important role in the development of hepatoma. Here, we designed four pairs of HBx-specific siRNAs on basis of the conserved region among the x gene and then examined whether the HBx-siRNAs with a lipid carrier can trigger immune responses in HepG2.2.15 cells. We then provided evidence that the non-specific innate immune responses induced by HBx-siRNAs were mediated by PKR. As type I IFN can be used for the treatment of virus hepatitis, these observations prompted us to investigate if the siRNA complex could achieve both gene silencing and IFN induction in hepatocytes. We finally demonstrate that the contribution of the immunostimulatory properties of siRNAs to HBV inhibition revealed in our study would be helpful for the siRNA-based antiviral therapy.

## Materials and Methods

### Cell line and cell culture

HepG2.2.15 cells (serotype ayw, genotype D), derived from HepG2 cells transfected with a plasmid carrying HBV genome DNA [20], were maintained in complete Dulbecco's modified Eagle medium (DMEM; GIBCO/BRL, USA) supplemented with 10% fetal bovine serum (FBS) at 37°C in a humidified atmosphere containing 5% CO_2_.

### Transfection

The sense and antisense strands of HBx-siRNAs were annealed and depurated by HPLC (RiboBio, Guangzhou China). HBx-siRNA sequences are shown in Table 1. HepG2.2.15 cells were seeded for 12 h, and transfected with Lipofectamine™ 2000/siRNA (Invitrogen Life Technologies, Carlsbad, CA, USA) according to the manufacturer's description. The PKR inhibitor C16 (Merck Calbiochem, Germany) was dissolved in DMSO according to the manufacturer's instructions and was added to cells at 1 h before exposure to siRNA, with the final concentration of 2 µM. For IFN Receptor (IFNR) neutralization, anti-IFNR antibody (PBL Biomedical Laboratories, USA) was added at 12 h before siRNA transfection [24]. The target sequences of PKR-siRNA (siPKR) were: GAA CUG CCU AAU UCA GGA C,and were synthesized by Ribo Company (RiboBio, Guangzhou, China). The Full-length human PKR expressing vector was generously provided by Stefan Rothenburg (Laboratory for Host-Specific Virology, Division of Biology, Kansas State University). The PKR catalytically inactive vector (cMyc-His-tagged PEF6-HPKR-K296R) was kindly supplied by BCCM/LMBP (Belgian Co-ordinated Collections of Micro-Organisms).

**Table 1:** Sequences of chemically synthesized HBx-siRNAs.

Name	Type	Sequence 5′→ 3′
siRNA1-sense	RNA	GGUCUUACAUAAGAGGACUdTdT
siRNA1-antisense	RNA	AGTCCTCTTATGTAAGACCdTdT
siRNA2-sense	RNA	GGACGUCCUUUGUUUACGUdTdT
siRNA2-antisense	RNA	ACGTAAACAAAGGACGTCCdTdT
siRNA3-sense	RNA	CCGACCUUGAGGCAUACUUdTdT
siRNA3-antisense	RNA	AAGUAUGCCUCAAGGUCGGdTdT
siRNA4-sense	RNA	UGUGCACUUCGCUUCACCUdTdT
siRNA4-antisense	RNA	AGGTGAAGCGAAGTGCACA dTdT

### Quantitative real-time polymerase chain reaction (PCR) analysis

Total RNA was prepared from treated HepG2.2.15 cells using Trizol extraction reagent (Invitrogen, Carlsbad, CA, USA). Approximately 2 µg of RNA was reverse transcribed using the M-MLV first-strand cDNA synthesis kit (Promega Corporation, Madison, WI, USA) and oligo(dT) primer as recommended by the manufacturer. Quantitative PCR was performed on an iCycleriQ real-time PCR system (Bio-Rad, USA). Amplified products were detected using SYBR Green PCR Master Mix (Toyobo Co. Ltd., Osaka, Japan). The sequences of primer pairs specific for each gene are shown in Table 2. The PCR was initially denatured at 95°C for 10 min, followed by 45 PCR cycles of 95°C for 30 s, 60°C for 30 s and 72°C for 30 s. The fold changes in expression were calculated relative to the expression of GAPDH.

**Table 2:** Sequences of primers specific for human genes used for real-time PCR analysis.

Target sequence	Sequence (5′ → 3′)	size (bp)
GAPDH	R: GAAGGTGAAGGTCGGAGTF: CATGGGTGGAATCATATTGGAA	155
IFN-α	R: CTC CTT TCT CCT GCC TGA AGF: AAG TGT CTC ATC CCA AGT AGC	170
IFN-β	R: TGCTCTCCTGTTGTGCTTCTCCF: CATCTCATAGATGGTCAATGCGG	222
ISG15	R: GGACAAATGCGACGAACCTCTF: CCCTCGAAGGTCAGCCAGA	158
ISG56	R: CTTGAGCCTCCTTGGGTTCGF: GCTGATATCTGGGTGCCTAAGG	137
iNOS	R: GACAAGAGGCTGCCCCCCF: GCTGGGAGTCATGGAGCCG	559
IL-6	R: CCACACAGACAGCCACTCACF: AGGTTGTTTTCTGCCAGTGC	146
IL-8	R: ACTTCCAAGCTGGCCGTGGCTCTCTTGGCAF: TGAATTCTCAGCCCTCTTCAA AAACTTCTC	295
TNF-α	R: ATCTTCTCGAACCCCGAGTGAF: CGGTTCAGCCACTGGAGCT	83
CXCL10	R: AGGAACCTCCAGTCTCAGCAF: CAAAATTGGCTTGCAGGAAT	193
CXCL2	R: TGCCAGTGCTTGCAGACF: TCTTAACCATGGGCGATGC	157
CCL4	R: CGCCTGCTGCTTTTCTTACACF: CAGACTTGCTTGCTTCTTTTGG	126
PKR	R: ATGATGGAAAGCGAACAAGGF: TTCTCTGGGCTTTTCTTCCA	76
HBV-X	R: CCGTCTGTGCCTTCTCATCTGCF: ACCAATTTATGCCTACAGCCTCC	256
HBV S/P	R: ATCCTGCTGCTATGCCTCATCTTF: ACAGTGGGGGAAAGCCCTACGAA	314

### Analysis of HBV DNA

Viral particles in the supernatants were quantified by fluorescence real-time polymerase chain reaction (FQ-PCR) according to the kit's instructions (Da-An, Guangzhou, China). Briefly, cells were transfected with siRNA as above. The supernatants were collected, and mixed with an equal volume of DNA extraction agent, then mixed violently for 10 s, centrifuged for several seconds and boiled for 10 min. Then, the supernatants were collected to test the content of HBV DNA. The primers specific for the HBV S region were P1: 5′-ATCCTGCTGCTATGCCTCATCTT-3′; P2: 5′-ACAGTGGGGGAAAGCCCTACGAA-3′. The fluorescent probe was 5′-RTGGCTAGTTTACAGTGCCATTTGQ-3′, R as report fluorophore and Q as quench fluorophore. The reaction was carried out for 40 cycles in the iCycleriQ real-time PCR system (Bio-Rad, USA).

### Flow cytometry

The cells were transfected with siRNA for 4 h. Intracellular detection of Stat-1 activation was determined by flow cytometry. Briefly, the cells were washed once before fixing in 0.4% paraformaldehyde in the dark at 4°C for 30 min. Cells were then washed twice in PBS before incubation with phycoerythrin (PE)-labeled antibody (Ab) to p-Stat-1 (BD Pharmingen, USA). Intracellular staining was performed in FACS buffer containing 0.01% saponin at 4°C for 1 h. Results were analyzed by FACS Calibur™ and CELL Quest™ software.

### Western blot analysis

HepG2.2.15 cells were lysed in lysing buffer [20 mM Tris–HCl, 150 mM NaCl, 1 mM Na_3_VO_4,_ 1 mM ethylenediaminetetraacetic acid (EDTA), 1 mM ethylene glycol tetraacetic acid (EGTA), 1 mM phenylmethylsulfonyl fluoride (PMSF), 50 mM NaF and 1% NP-40]. After SDS-PAGE, proteins were transferred to polyvinylidene fluoride (PVDF) membranes and then incubated with the following specific antibodies: mouse anti-human HBx mAb (Millipore, USA), rabbit anti-human-PKR and anti-p-PKR mAb, rabbit anti-human-eIF2-α and anti-p-eIF2-α mAb (Santa Cruz, CA, USA), or anti-human-β-actin mAb (Cell Signaling Technology, New England BioLabs Inc., USA) diluted in 1∶1000 at 4°C. After washing with Tris-buffered saline-Tween 20 (TBST) three times, the membranes were incubated with horseradish peroxidase (HRP)-conjugated second antibody. Protein bands were visualized using Anmobilon™ Western chemiluminescent HRP Substrate system (Millipore Corporation. Billerica, Massachusetts, USA).

### ELISA

The cells were treated as above. Supernatants were harvested after 24 h or 48 h stimulation with siRNA (150 nM). The level of IFNα was quantified using sandwich ELISA kits from R&D Systems. The levels of HBsAg and HBeAg were detected by ELISA kit from Rong Sheng (Shanghai, China).

### Immunocytochemical staining

HepG2.2.15 cells were grown on glass cover slips in a 24-well plate at a density of 5×10^4^ cells per well. Then, the cells were transfected with HBx-siRNAs. After 48 h, the cells were fixed in 4% paraformaldehyde for 10 min, and permeabilized with 0.2% Triton X-100 for 30 min. Then cells were exposed to 3% H_2_O_2_ for 10 min at 25°C and blocked in 5% bovine serum albumin (BSA) for 30 min. The cells were incubated with anti-core antigen (HBcAg) antibody (Genetech company, Shanghai, China) at 4°C overnight, washed, and incubated with appropriate biotin-conjugated secondary antibody for 10 min followed by incubation with streptavidin-peroxidase for 10 min and 3,3′-diaminobenzidine (DAB) detection. The staining was visualized by light microscopy (Olympus).

### Data analyses

Data are expressed as mean ± SD for three or more independent experiments. Statistical differences between any two groups were determined by *t*-test. *p*-values <0.05 were considered statistically signficant.

## Results

### HBx-siRNAs inhibit HBV expression in HepG2.2.15 cells

**Figure 1:**
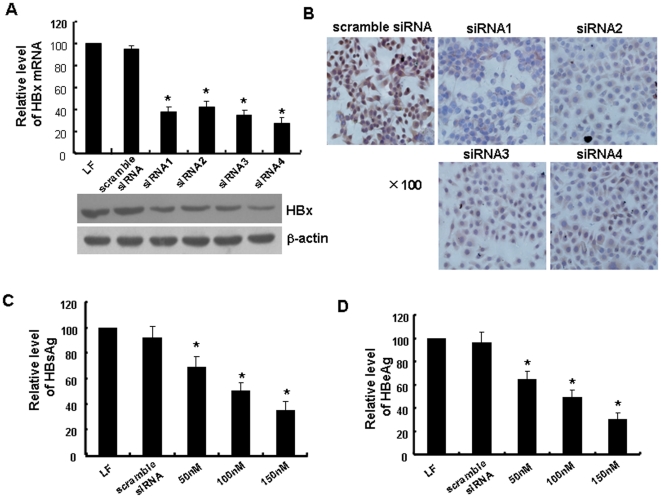
HBx-siRNA inhibits hepatitis B virus (HBV) expression in HepG2.2.15 cells. (A) Scramble siRNA or HBx-siRNA (siRNA1, siRNA2, siRNA3, siRNA4) (100 nM) was transfected into HepG2.2.15 cells using lipofectamine 2000 (LF). LF-treated group was used as a control. The cells were harvested, and RNA was extracted at 24 h after transfection. Expression of HBx mRNA were detected by real-time PCR (top). The level of each sample is expressed as the percentage of the RNA level in scramble siRNA-treated cells. The levels of HBx protein were detected by western blot at 48 h after transfection (bottom). (B) HepG2.2.15 cells were transfected for 48 h, and then cells were fixed, immunostained for HBcAg and visualized under a microscope. Representative fields from each cell group are shown (original magnification ×100). (C, D) The supernatants were harvested after 48 h, and the dose-dependent reductions in HBsAg (C) and HBeAg (D) were detected with ELISA. Data showing the silencing effect are representative of three independent experiments. Data are expressed as the mean ± SD from at least three separate experiments. *p<0.05 versus the LF-treated group.

In order to test the capability of HBx-siRNAs to inhibit HBV replication in HepG2.2.15 cells, we delivered HBx-siRNAs into HepG2.2.15 cells using Lipofectamine™ 2000. Then HBx expression was detected with quantitative RT-PCR at 24 h after transfection with the four HBx-siRNAs. We found that treatment with HBx-siRNAs resulted in decrease in HBx expression by 61.7±2.1%, 57.5±4.3%, 62.5±2.5%, and 72.6±3.1% (*p*<0.05) respectively, in the total mRNA at a concentration of 100 nM, compared with the LF-treated cells (Figure 1A, top). Western blot analysis also showed a significant silencing effect of HBx-siRNAs on HBx expression at protein level (Figure 1A, bottom).

Immunohistochemical staining for HBcAg in siRNA-treated HepG2.2.15 cells allows us to directly observe the HBV clearance, as the presence of cytoplasmic HBcAg is an indicator of active viral replication. As shown in Figure 1B, cytoplasmic HBcAg staining in HBx-siRNA-treated HepG2.2.15 cells decreased significantly at 48 h after transfection. Levels of HBsAg and HBeAg were also inhibited significantly in a dose-dependent manner (Figure 1C and 1D). HBsAg secretion was reduced by 69.5±6.3% when siRNA4 was transfected at 150 nM. The scramble siRNA exhibited no suppressive effect. These results demonstrated that HBx-siRNAs not only silenced the expression of HBx gene, but also inhibited the expression of HBcAg and secretion of HBsAg and HBeAg.

### Innate immune responses induced by HBx-siRNAs in HepG2.2.15 cells

**Figure 2:**
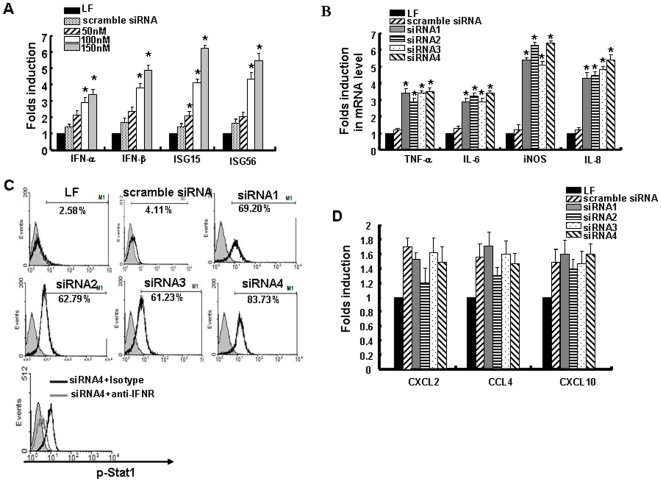
HBx-siRNA induced the expression of type I interferon (IFN) and inflammatory cytokines in HepG2.2.15 cells. (A) HepG2.2.15 cells were transfected with HBx-siRNAs; 24 h later, the mRNA levels of IFN-α, IFN-β, ISG15 and ISG56 were measured by real-time PCR. The mRNA level in each sample was expressed as a fold change of the RNA level in lipofectamine (LF)-treated cells. The levels of mRNA are presented relative to mock control. (B) Cells were transfected with HBx-siRNAs for 24 h. Levels of TNF-α, IL-6, iNOS, and IL-8 mRNA were analyzed by real-time PCR and presented relative to those of LF-treated cells. (C) HepG2.2.15 cells were incubated with phycoerythrin (PE)-labelled anti-p-Stat1 antibody after HBx-siRNA transfection for 4 h, and were then analyzed by flow cytometry. p-Stat1 expression was also detected when IFN receptor was neutralized. (D) Levels of CXCL2, CCL4, and CXCL10 mRNA were analyzed by real-time PCR and presented relative to those of LF-treated cells. Data are expressed as the mean ± SD from at least three separate experiments. *p<0.05 versus LF-treated group.

The expression profile of several antiviral molecules was investigated after HBx-siRNA transfection into HepG2.2.15. Synthesis of mRNA for IFNα, IFNβ, and interferon-stimulated genes (ISG) were significantly induced as a consequence of HBx-siRNA transfection, while scramble siRNA (100nM) has a weak induction of IFN and ISGs (Figure 2). IFNα expression showed a 3.4-fold increase at 24 h after siRNA4 transfection at a concentration of 150 nM when compared with mock-treated cells. Moreover, induction of IFN responses is in a concentration-dependent manner (Figure 2A).

Recent experiments have also indicated that interleukin (IL)-6 suppresses HBV replication in an HBV-producing cell line [25]. Our results showed that the HBx-specific siRNAs induced mRNA expression of tumor necrosis factor (TNF)-α and IL-6 up to 3.6-fold relative to lipofectamine treatment in HepG2.2.15 cell lines (*p*<0.05) (Figure 2B). The mRNA levels of IL-8 and inducible nitric oxide synthase (iNOS) were also enhanced at 24 h post-transfection. Scramble siRNA that was used as a negative control was not capable of inducing expression of IL-8, IL-6, iNOS, and TNF-α in HepG2.2.15 cells (Figure 2B). Similar results were also observed in both HepG2 and HL-7702 cell lines (data not shown).

We further detected phosphorylation of Stat1, a molecule downstream of the type I IFN signaling pathway. Flow cytometry revealed a signficantly higher level of p-Stat1 expression in HepG2.2.15 cells that had been transfected by HBx-siRNAs for 4 h (Figure 2C). In order to see if Stat1 phosphorylation is due to IFN induction, we blocked the type I IFN pathway with a neutralizing antibody against the IFN-α/β receptor. The results showed that p-Stat1 activation was suppressed after IFN receptor neutralization (Figure 2C, bottom). The expression of inflammatory-related chemotactic factors, such as CXCL2, CCL4 and CXCL10, did not change signficantly after HBx-siRNA transfection (Figure 2D). These results demonstrated that HBx-siRNAs induced the expression of type I IFN and related genes as well as inflammatory cytokines in HepG2.2.15 cells.

### HBx-siRNAs promote the activation of PKR and its downstream signal pathway

**Figure 3:**
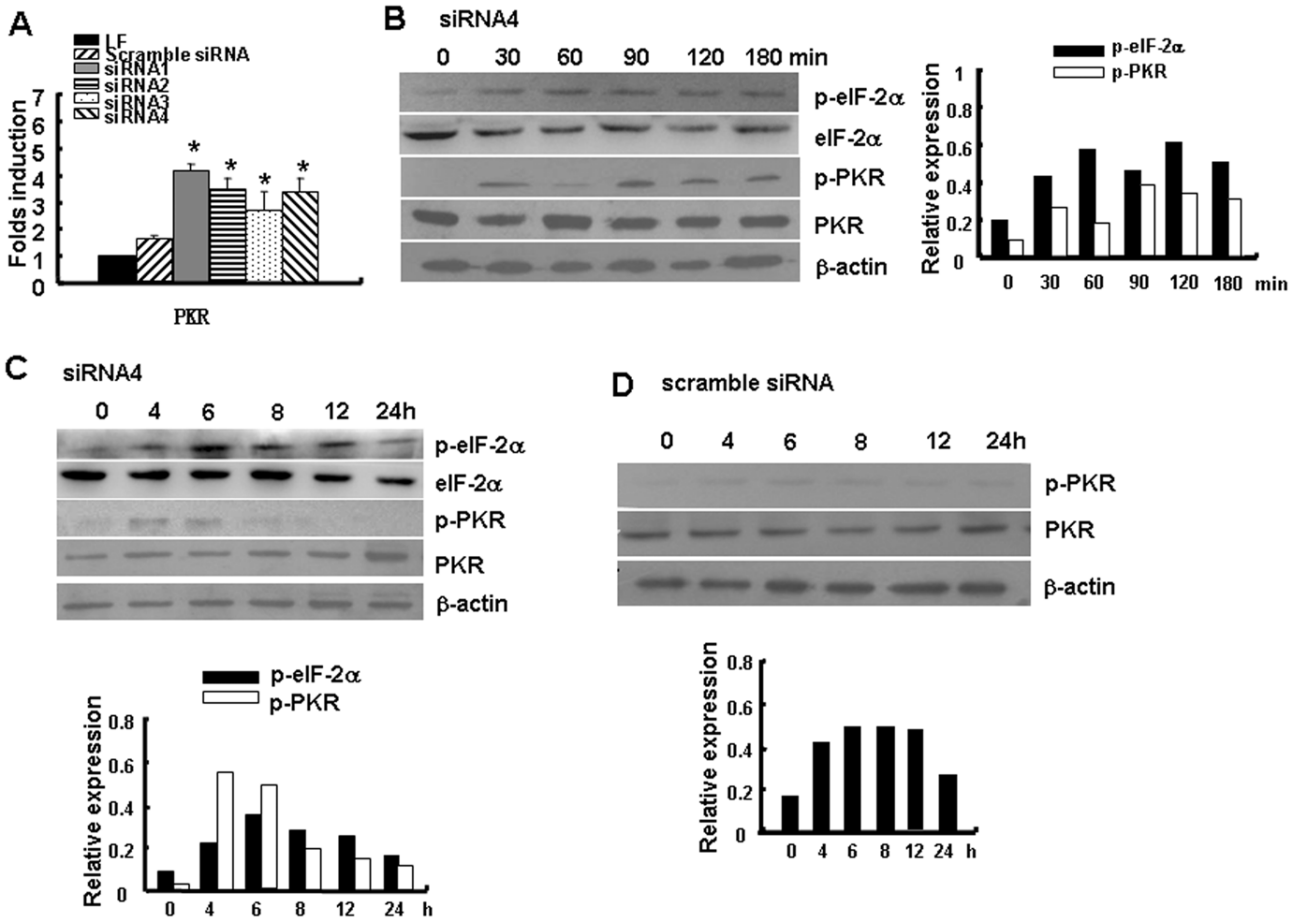
HBx-siRNA promoted the expression of PKR and the activation of PKR signaling pathway. (A**)** Real-time PCR was used to analyze the mRNA expression of PKR after HBx-siRNAs treatment for 24 h. The mRNA level in each sample was expressed as fold change compared with RNA level in LF-treated cells. Data are expressed as the mean ± SD from at least three separate experiments. *p<0.05 versus LF-treated group. (B) Western blot analysis of the activation of the protein kinase R (PKR) and PKR substrate, translation initiation factor 2 (eIF-2)α, from HepG2.2.15 cells. Cells were transfected with siRNA4 (150 nM) at different time points (0, 30, 60, 90, 120, and 180 min) using lipofectamine. The panel used an antibody specific to whole PKR or eIF2-α that demonstrates equal loading of total protein. Histogram showed the relative expression of p-PKR or p-eIF2-α after normalization to whole PKR or eIF2-α (right). (C) Western blot analysis of the activation of the PKR and eIF2-α by siRNA4 at different time points (0, 4, 6, 8, 12, and 24 h). Histogram showed the relative expression of p-PKR or p-eIF2-α (bottom). (D) Western blot analysis of the activation of the PKR by scramble siRNA at different time points (0, 4, 6, 8, 12, and 24 h). Histogram showed the relative expression of p-PKR (bottom).

Several reports have demonstrated that siRNA can induce IFN responses in immune and non-immune cells and that these responses are based on the recognition of cytoplasmic dsRNA as a signature of viral infection [15], [23], [24], [25]. PKR activation is found to be involved in the innate immune responses induced by siRNA [19]. To explore the possible mechanism of HBx-siRNA-mediated IFN induction, the expression level of PKR was examined. RT-PCR analysis demonstrated that transfection of HepG2.2.15 cells with HBx-siRNA did increase the expression of PKR at the mRNA level (Figure 3A). Western blot analysis demonstrated that transfection with HBx-siRNA promoted the phosphorylation of PKR, and that the PKR substrate eIF2-α was also phosphorylated (Figure 3B). We further performed a longer kinetics over a few hours (0, 4, 6, 8, 12 and 24 h) and found that HBx-siRNA promoted the phosphorylation of PKR until 6 h and enhanced the activation of eIF2-α until 12 h post-transfection (Figure 3C), while the scramble siRNA showed a weak activation of PKR (Figure 3D). These results demonstrated that PKR might be involved in the immune responses induced by HBx-siRNA in HepG2.2.15 cells.

### PKR is involved in the innate immune responses induced by HBx-siRNAs in HepG2.2.15 cells

**Figure 4:**
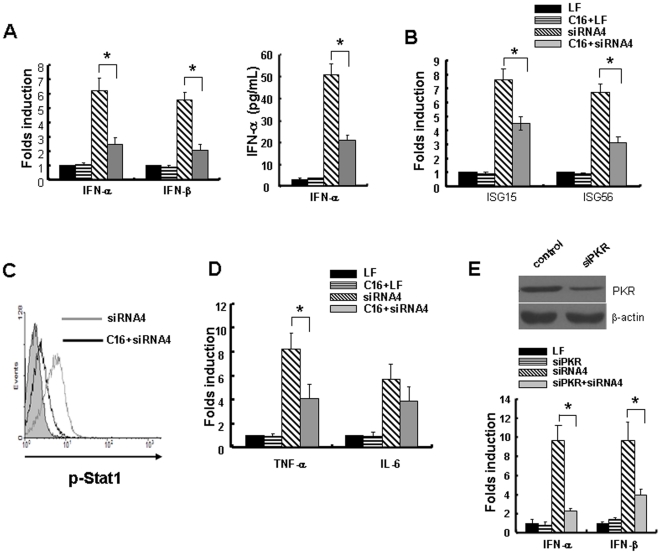
Inhibition of protein kinase R (PKR) activity attenuated HBx-siRNA-induced innate immune responses. (A) HepG2.2.15 cells were treated with or without PKR inhibitor C16, and then transfection experiments were performed for 24 h. Levels of IFN-α and IFN-β mRNA were analyzed by real-time PCR and presented relative to mock transfection (left). The levels of IFN-α in supernatants were examined by ELISA (right). (B) The experiment was performed as Fig. 4A. Levels of IFN-stimulated gene (ISG)15 and ISG56 mRNA were analyzed by real-time PCR and presented relative to mock transfection. (C) p-Stat1 expression was detected by flow cytometry when siRNA was transfected for 4 h. (D) The experiment was performed as Fig. 4A, mRNA levels of TNF-α and IL-6 were analyzed by quantitative real-time PCR and were presented relative to mock transfection. (E) siPKR were transfected into cells, then PKR protein expression was assayed by Western Blot (top). siRNA4 and siRNA targeting PKR were cotransfected into HepG2.2.15 cells for 24 h, then mRNA levels of IFN-α and IFN-β were analyzed and presented relative to mock transfection (bottom). Data are expressed as the mean ± SD from at least three separate experiments. *p<0.05 versus siRNA4-treated group.

To find out if PKR is indeed responsible for the immunomodulatory effects of HBx-siRNAs, we first treated HepG2.2.15 cells with PKR specific inhibitor C16 and then transfected cells with HBx-siRNA4. We found that the induction of IFN-α and IFN-β mRNA as well as the expression of IFN-stimulated genes ISG15 and ISG56 were attenuated significantly after HBx-siRNA transfection (Figure 4A and 4B). p-Stat1 activation was also suppressed as assayed by flow cytometry (Figure 4C). In addition, proinflammatory factors TNF-α and IL-6 were also decreased when C16 was added (Figure 4D). We then cotransfected siRNA targeting PKR (siPKR) and HBx-siRNA in HepG2.2.15 cells to further determine the involvement of PKR in the innate immune responses. As shown in Figure 4E, siPKR decreased PKR protein level (top) and significantly attenuated the induction of IFN-α and IFN-β (bottom).

**Figure 5:**
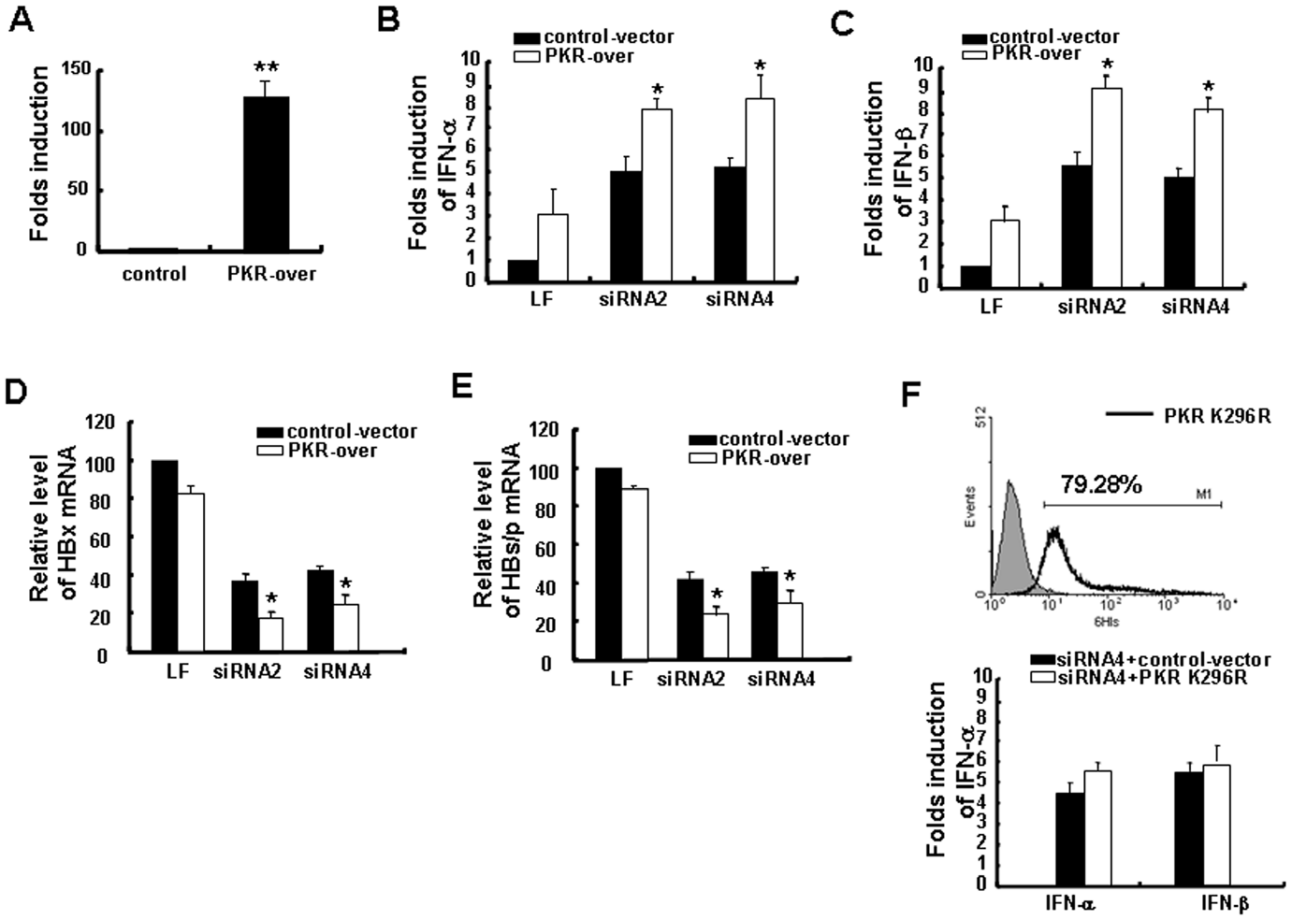
Protein kinase R (PKR) over-expression enhanced type I interferon (IFN) production and HBx-siRNA-mediated hepatitis B virus (HBV) inhibition. HepG2.2.15 cells were transfected with 2 µg of full-length PKR plasmid or the control plasmid. 24 h later, the cells were transfected with HBx-siRNA (150 nM). (A) Over-expression of PKR at the transcriptional level. (B, C) Levels of IFN-α and IFN-β were detected by real-time PCR at 24 h. (D, E) mRNA expression levels for HBx and HBs/p were detected by real-time RT-PCR at 24 h. (F) HepG2.2.15 cells were transfected with 1 µg of hPKR-K296R or the control plasmid. Then cells were collected and 6-His was assayed by FACS (top). When transfected with hPKR-K296R for 24 h, the cells were transfected with HBx-siRNA4 (150 nM). Another 24 h later, levels of IFN-α and IFN-β were detected. The levels of all examined parameters were expressed as percentages of the corresponding parameter in LF-treated control-vector transfected cells (bottom). Data are expressed as the mean ± SD from at least three independent experiments. *p<0.05 versus corresponding control-vector transfected cells.

Furthermore, HepG2.2.15 cells were first transfected with full-length PKR plasmids followed by HBx-siRNA transfection. As illustrated in Figure 5A, PKR was successfully over-expressed in HepG2.2.15 cell line at the transcriptional level. We found that the mRNA levels of IFN-α and IFN-β in PKR-overexpression cells were increased more markedly when stimulated with HBx-siRNA than that in control vector-transfected cells (Figure 5B, 5C). Furthermore, HepG2.2.15 cells were transfected with a PKR catalytically inactive vector (hPKR-K296R) [26]. As the PKR-K296R plasmid contains the C-terminal Myc epitope and polyhistidine tag (His-tag), we assayed 6His expression by FACS to detect the PKR-K296R expression. The results showed that PKR-K296R has been successfully expressed (Figure 5F, top). We found that PKR-K296R overexpression could not further promote type I IFN production induced by HBx-siRNA as full-length PKR (wide type) did (Figure 5F, bottom). These results suggest that the promotion of PKR in IFN induction mediated by HBx-siRNA is dependent on the catalytic activity of PKR. In general, the results above confirm that PKR activation indeed contributes to the innate immune responses induced by HBx-siRNAs.

### PKR activation contributes to HBV inhibition in HepG2.2.15 cells

**Figure 6:**
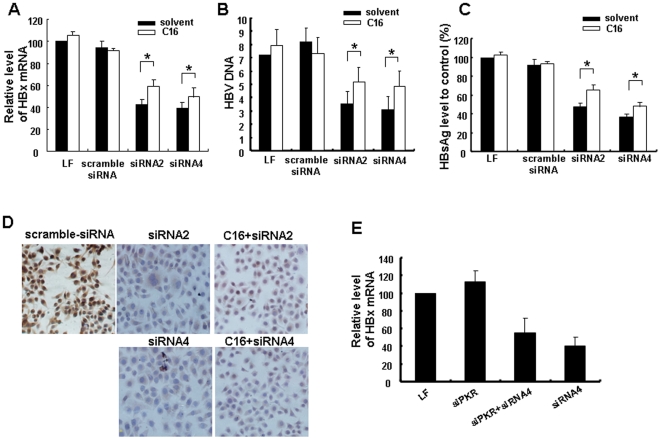
Inhibition of protein kinase R (PKR) activity attenuated HBx-siRNA-induced hepatitis B virus (HBV) inhibition. HepG2.2.15 cells were treated with or without C16, and then transfection experiments were performed at a concentration of 150 nM. Levels of HBx RNA mRNA **(**A) and HBsAg proteins in supernatants (C) were examined as described previously. Levels were expressed as percentages of corresponding levels in scramble siRNA-treated cells. HBV DNA in supernatants was detected by real-time PCR (B). Immunostaining for HBcAg were visualized under a microscope (D). siRNA4 and siPKR were cotransfected into HepG2.2.15 cells, then levels of HBx mRNA were analyzed by quantitative real-time PCR and presented relative to mock transfection (E). Data are expressed as the mean ± SD from at least three independent experiments. *p<0.05 versus corresponding solvent-treated group.

To determine if immune stimulation induced by HBx-siRNAs contributes to the inhibition of HBV, HepG2.2.15 cells were treated with C16 to inhibit PKR activity before HBx-siRNA transfection. As shown in Figure 6, C16 strongly attenuated the effects of HBx-siRNAs in HBV inhibition. Transfection of HBx-siRNAs resulted in 63.2, 68.5, 69.6, and 65.8% suppression of HBx gene expression respectively compared with the solvent treatment group. Treatment with C16 significantly attenuated the suppression of HBx expression (Figure 6A). The inhibition of HBV DNA, HBsAg or HBcAg was also attenuated (Figure 6B–6D). We further knocked down PKR expression in HepG2.2.15 cells by siPKR, and found that siPKR also significantly attenuated the HBx inhibition by HBx-siRNA (Figure 6E).

To further determine if the observed up-regulation of PKR leads to a reduction in HBV apart from the specific inhibition of HBV through RNAi pathway, we transfected HepG2.2.15 cells with plasmid containing full-length PKR, and then assessed the changes in HBV expression. As shown in Figure 5D and 5E, the levels of HBx and HBs/p were decreased more obviously in cells with over-expression of PKR. These results indicated that over-expression of PKR further increased the inhibition of HBV expression by HBx-siRNAs and that recognition and activation of PKR were involved in and promoted the inhibition of HBV expression by siRNA. In conclusion, our results suggested that HBx-siRNAs-induced innate immune responses play important roles in HBV inhibition and clearance in addition to the direct RNA silencing by RNAi.

## Discussion

This study and earlier studies have shown that RNAi can be successfully used for HBV inhibition. In addition, we report that: (1) HBx-siRNA can induce innate immune response associated with the release of type I IFN and related cytokines in HepG2.2.15 cells; (2) this effect does boost silencing of specific targets; and (3) these innate immune stimulatory effects of lipid-siRNA in HepG2.2.15 cells are at least partly mediated by the cytoplasmic kinase, PKR.

Studies have demonstrated that RNA can be recognized by PRRs, including PKR, TLR3, TLR7, TLR8, RIG-I and MDA-5 [19], [21], [23], [27], [28]. PKR is an important cytoplasmic sensor of viral infection that can be activated by dsRNA. During viral infection, PKR can phosporylate eIF2-α and activate the NF-κB pathway for regulation of cytokine expression and the inflammatory response [19], [20], [21]. The RNA-binding sites of PKR contain two dsRNA-binding motifs (dsRBMs) linked by 20 amino acids. Previous studies have suggested that PKR required at least 30 bp of dsRNA for efficient activation, whereas another study demonstrated that as little as 17 bp of dsRNA could activate PKR signal [29], [30]. Recent studies have reported that siRNA induces non-specific immunostimulatory effects in non-immune cells, such as epidermal keratinocytes, breast cancer cell MCF-7, glioma cell T98G, mouse embryo fibroblasts MEFs and renal cell carcinoma [19], [21], [23], [31]. Here, for the first time, we found the up-regulation of type I IFN and related genes as well as inflammatory cytokines, the increased expression of PKR, and the phosphorylation of eIF2-α in HepG2.2.15 cells after exposure to HBx-siRNAs. Inhibition of PKR activity by PKR inhibitor C16 or silencing of PKR expression by RNAi significantly attenuated these effects, while PKR overexpression further promoted IFN responses and HBV inhibition. Our results demonstrated that HBx-siRNAs activated innate immune responses in hepatoma cells in a PKR-dependent manner. We attribute this HBV inhibition to the combination of RNAi and immune stimulation, which may arise from the production of type I IFN and other antiviral genes. In addition, we found that HBx-siRNAs transfection also increased TLR3 expression significantly and promoted RIG-I expression slightly in HepG2.2.15 cells (data not shown). We propose that TLR3 and RIG-I recognition might also contribute to the activation of innate immune responses induced by HBx-siRNAs, which need to be further determined. It is well known that TLRs can respond to RNA molecules and play crucial roles in the host defense against viral infection. In plasmacytoid dendritic cells, siRNA induces IFN-α secretion in a sequence-dependent manner through TLR7 [28]. Here, in HepG2.2.15 cells, we observed that HBx-siRNAs transfection neither stimulated TLR7 expression, nor activated the TLR7 signaling pathway (data not shown), thus suggesting that TLR7 is not involved in HBx-siRNA recognition in HepG2.2.15 cells.

As a direct evidence of PKR activation, eIF2-α phosphorylation is thought to inhibit translation of most cell and viral mRNAs [32]. However, some reports have found that it do not affect the overall translation of the cell mRNAs [33]. In contrast, translation of some mRNAs is enhanced after eIF2-α phosphorylation. For example, activation of PKR can induce the expression of Fas and trigger apoptosis through the FADD/caspase-8 death signaling pathway [34], [35]. For type I IFN, it has been found that PKR activation promotes the production of autocrine IFN, in addition to inhibiting the translation of viral mRNAs through phosphorylation of eIF2-α [36]. Indeed, we show in the present study that PKR activation by HBx-siRNA increased production of IFN-α and IFN-β both at mRNA and protein level (Figure 4A). The mechanisms is probably that PKR activation transmits signals not only to eIF2-α and the translational machinery but also to various factors such as STAT, interferon regulatory factor 1 (IRF-1), as well as engaging the NF-κB pathway.

Accumulating data have shown that HBV products inhibit type I IFN production through suppressing TLR- or RIG-I-madiated innate immune signal pathway [37], [38], [39]. Recent data suggest that HBV-bearing supernatants, purified HBV virions, and recombinant HBsAg or HBeAg can suppress the innate immune response elicited by TLR stimulation in hepatocytes and nonparenchymal liver cells, leading to suppression of IRF-3 activation and down-regulation of IFN-β production [38], [40]. HBx, acting as an inhibitor of TLR-triggered induction of type I IFNs, suppresses MxA expression at the promoter level and inhibits cellular proteasome activities [39], [41], [42]. Our recent finding and other research show that HBV, particular HBx, inhibits RIG-I activation and down-regulates production of type I IFN [41], [42], [43]. Therefore, we concluded that the type I IFN induction in response to HBx-siRNAs probablly derived from two aspects: on one hand, HBx-siRNAs stimulate PKR activation and then induce IFN-α or IFN-β production;on the other hand, HBx-siRNAs relieve the inhibition of innate immune response mediated by HBV.

The present study showed that HBx-siRNA-induced PKR activation contributes to HBV inhibition and this dual function of HBx-siRNAs in both inhibiting HBV replication and triggering innate immunity in a PKR-dependent manner would be beneficial for HBV clearance. In addition to type I IFN, levels of inflammatory cytokines TNF-α, IL-6, and IL-8 were also increased significantly upon PKR activation by HBx-siRNA (Figure 2B), we believed that they contributes to the suppression of HBV replication, as reported by others that inflammatory cytokines, esp. IL-6, control HBV gene expression in HBV infection [25], [44]. Future work will focus on *in vivo* immune modulation strategies of these dual functional HBx-siRNAs available to inhibit HBV.

In conclusion, this study demonstrates that HepG2.2.15 cells can recognize siRNA and develop non-specific innate immune responses through intracellular kinase PKR and that the induction of innate responses facilitates the effects of HBV inhibition. In addition to HBV, other virus-related siRNAs, such as siRNA targeting respiratory syncytial virus NS1 (siNS1) and siRNA targeting human papillomavirus (HPV), are also reported to induce innate immune responses by upregulating expression of IFN-β and IFN-inducible genes [45], [46]. Understanding and controlling the activation of the immune response is an important step toward using siRNA molecules therapeutically. The combination of RNAi and immune stimulation may be beneficial for treatment of HBV and other infectious virus diseases, raising concerns about clinical trials of systemically delivered siRNAs.
